# Global burden of anemia attributable to non-communicable diseases: GBD 2021 analysis and projections

**DOI:** 10.3389/fnut.2025.1557986

**Published:** 2025-08-06

**Authors:** Danqing Yu, Yulu Ni, Kai Chen, Haiyan Xu, Xin Huang, Yinhui He

**Affiliations:** Department of Endocrinology and Metabolism, The Fifth Affiliated Hospital of Wenzhou Medical University, Lishui Municipal Central Hospital, Lishui, China

**Keywords:** anemia, non-communicable diseases (NCDs), years lived with disability (YLDs), socio-demographic index (SDI), Global Burden of Diseases (GBD), ARIMA model

## Abstract

**Background:**

Anemia is a significant global health issue affecting approximately one-third of the world’s population. Non-communicable diseases (NCDs) such as diabetes, cardiovascular diseases, and chronic kidney disease are increasingly recognized as significant contributors to anemia, particularly in populations with high prevalence of these conditions.

**Methods and results:**

Globally, the prevalence of anemia caused by NCDs has increased significantly from 249.1 million to 368.4 million, representing a 48% increase since 1990. A similar trend can be observed in YLDs. The burden was expected to continue to grow in the next 20 years. The curves of anemia-related burden showed an intersection for different genders around the age of children under five and women of reproductive age, beyond which women exhibit a higher burden of anemia, compared to men. Populous countries like India [102.35 million (95% UI: 97.49–108.13)], and China [45.63 million (95% UI: 42.15–49.89)] have the largest number of anemia cases caused by NCDs. Hemoglobinopathies and hemolytic anemias, being the key underlying cause of anemia, accounted for 69.0% of the anemia-related burden. There is a discernible upward trend across all socio-demographic index (SDI) groups over the years, particularly in low-middle SDI countries. While age-standardized, the burden of anemia in all SDI groups has shown a decrease trend. And the highest prevalence rates and YLDs rates always appeared in low-middle and middle SDI regions, similar with that of health system grouping levels.

**Conclusion and interpretation:**

Our study reveals that anemia is particularly prevalent in low- and middle-income countries, where it significantly impacts vulnerable populations such as children under five and women of reproductive age. Understanding these multifaceted influences is essential for developing targeted and effective strategies to alleviate the burden of anemia globally.

## Introduction

Anemia, defined as a condition characterized by a reduction in the number of red blood cells or the amount of hemoglobin, is a significant global health issue affecting approximately one-third of the world’s population (1). The World Health Organization (WHO) sets specific hemoglobin concentration thresholds to diagnose anemia, which vary by age, sex, and pregnancy status. The etiology of anemia is multifactorial, with nutritional deficiencies such as iron, vitamin B12, and folate being the most common causes (2). Other significant contributors include chronic diseases, infections like malaria and tuberculosis, and genetic disorders such as thalassemia and sickle cell disease. Non-communicable diseases (NCDs) such as diabetes, cardiovascular diseases, and chronic kidney disease are increasingly recognized as significant contributors to anemia, particularly in populations with high prevalence of these conditions. The relationship between NCDs and anemia is multifaceted, with chronic inflammation and impaired erythropoiesis being key mechanisms linking these conditions (3).

Epidemiologically, anemia is particularly prevalent in low- and middle-income countries, where it significantly impacts vulnerable populations such as children under five and women of reproductive age (4). The Global Burden of Disease Study indicates that anemia contributes to a substantial number of years lived with disability and is associated with increased morbidity and mortality rates. In 2010, anemia accounted for 68.4 million years of life lived with disability, or 9% of the total global disability burden (5).

The economic burden of anemia is substantial, impacting both individuals and healthcare systems. It leads to reduced productivity and increased healthcare costs, further exacerbating the economic disparities in low- and middle-income countries. The socioeconomic burden of anemia is considerable, affecting not only individual health but also economic productivity (6). In adults, anemia can lead to reduced work capacity, while in children, it impairs cognitive and physical development. Interventions aimed at addressing anemia must consider the complex interplay of nutritional deficiencies, chronic diseases, and genetic factors (7). Nutritional interventions, such as iron and vitamin supplementation, remain crucial, but their effectiveness varies depending on the underlying causes of anemia (8).

Gender and geographical disparities in the burden of anemia are evident. Females are consistently at greater risk of anemia than males across almost all geographic regions and in most age groups (9, 10). Geographically, sub-Saharan Africa, South Asia, and the Caribbean have the highest prevalence of anemia. Economic development and cultural practices play a crucial role in shaping the anemia landscape. Improved economic conditions can enhance access to nutritious food and healthcare, thereby reducing anemia rates. However, cultural practices, such as dietary habits and traditional beliefs, can either mitigate or exacerbate the risk of anemia, depending on their alignment with nutritional requirements. Understanding these multifaceted influences is essential for developing targeted and effective strategies to alleviate the burden of anemia globally (9, 11, 12).

Nevertheless, comprehensive analyses of the global prevalence and years lived with disability (YLDs) for anemia over extended periods, considering regional and socioeconomic disparities, are relatively scarce. Additionally, there is a dearth of research documenting the proportional impact of anemia across various etiologies and genders. This underscores the necessity to investigate the trends in anemia-related burden attributable to non-communicable diseases (NCDs) to inform health promotion and disease prevention initiatives. In response to these gaps, this study seeks to provide a thorough evaluation of the burden of anemia resulting from NCDs, considering temporal, national, age, gender, and socioeconomic dimensions, and to project these trends up to 2045. The goal is to offer valuable insights for the formulation of targeted health policies.

## Method

### Data collection

The Global Burden of Disease Collaborative Network offers extensive epidemiological data encompassing 288 causes of death, 371 diseases and injuries, and 88 risk factors across 204 countries and territories from 1990 to 2021. This dataset is derived from the GBD 2021 and is available for public use, thus negating the requirement for informed consent and ethical approval. The data can be accessed at the following URL: https://vizhub.healthdata.org/gbd-results/.

### Case definitions and input data

Anemia Attributable to NCDs: “Defined as hemoglobin levels <130 g/L (men) or <120 g/L (women) where the primary etiology was linked to GBD-coded non-communicable diseases (e.g., CKD, IBD, malignancies), excluding nutritional/deficiency causes. To account for variations in diagnostic criteria, adjustments were applied to align heterogeneous data sources. Sequelae, including neuropathy, vision impairment, and amputations, were categorized using ICD-10 codes (D63).

Within the Global Burden of Disease 2021 (GBD 2021) framework, anemia was characterized by reduced hemoglobin (Hb) levels in the bloodstream, regardless of etiology, erythrocyte morphology, or function. Diagnostic criteria for anemia presence and severity adhered to WHO-established hemoglobin thresholds (g/L) (13). Notably, distinct hemoglobin cut-offs were applied to neonates compared to other children under 5 years old. Given the absence of international neonatal anemia guidelines, thresholds combined WHO recommendations (for infants 6–59 months) with typical newborn hemoglobin concentrations (14). Definitions for mild, moderate, and severe anemia based on hemoglobin levels are detailed in Supplementary Table S1.

Estimating total anemia prevalence utilized diverse data sources. Inclusion required quantitative hemoglobin measurement from population-representative samples or groups reflecting the study’s age, sex, and location demographics. Primary data originated from population surveys, including the Demographic and Health Survey (DHS), Multiple Indicator Cluster Survey (MICS), national micronutrient assessments, and other nutritional surveys at national/sub-national levels. These were augmented by records from the WHO Vitamin and Mineral Nutrition Information System (VMNIS).

Sources containing individual-level data were aggregated into GBD age-sex groups. Data extraction followed seven criteria: mean Hb, Hb standard deviation (SD), prevalence of severe anemia, moderate anemia, combined moderate/severe anemia, mild anemia, and total anemia prevalence. Pregnancy status was documented without Hb value adjustments. Mean Hb and SD values were obtained from VMNIS and literature where possible. Sources lacking mean/SD data were excluded from modeling but contributed prevalence data for model validation. The analysis incorporated 703 distinct data sources spanning 153 countries.

Primary epidemiological sources encompassed population-based surveys (e.g., DHS, MICS), national micronutrient studies, VMNIS-compiled records, and other nutrition surveys. Comprehensive documentation of Global Burden of Disease Study 2021 data resources is accessible through the GHDx repository at http://ghdx.healthdata.org/gbd-2021/data-input-sources. Variations in regional database types were noted.

### Data handling and modeling approach

Most surveys employed the HemoCue test, modified for elevation, while excluding individuals with terminal or acute illnesses. Conversely, scientific literature and higher-income settings typically utilized Coulter counters for hemoglobin quantification. Both methodologies, considered equivalent in this analysis, function by reacting hemoglobin with Drabkin’s reagent and measuring light absorbance. Studies employing either venous whole blood or capillary sampling were treated identically (14).

Given hemoglobin concentration elevation at higher altitudes, values were corrected using WHO-recommended formulas. Sources providing pre-adjusted Hb data were incorporated directly; those reporting unadjusted values alongside altitude data underwent correction using this formula (13, 14). All included studies underwent age and sex disaggregation to align with predefined GBD demographic categories.

Pregnancy-related Hb data processing varied by source. Population-based studies sampling pregnant women followed criteria in Supplementary Table S1 without Hb modification, assuming pregnancy rates reflected the surveyed adult female population. Studies exclusively involving pregnant subjects were statistically mapped to the general population via the meta-regression–Bayesian, regularized, trimmed (MR-BRT) technique (14).

### Outcome compilation

Disability weights (DWs) derived from the GBD 2013 European study were applied: mild [0.004 (0.001–0.008)], moderate [0.052 (0.034–0.076)], severe [0.149 (0.101–0.209)] anemia (15). Years Lived with Disability (YLDs) were computed by multiplying category-specific prevalence by corresponding DWs.

Since anemia-attributable mortality was absent, Years of Life Lost (YLLs) equaled zero; thus, Disability-Adjusted Life Years (DALYs) equated to YLDs. Age-standardized rates (per 100,000 population) were calculated for point prevalence and YLDs using the GBD reference population. Uncertainty propagation involved 1,000 sample draws per computational phase, incorporating error sources from input data, measurement corrections, and residual non-sampling error. Uncertainty intervals (UIs) represent the 2.5th and 97.5th percentiles of ordered draws. Smoothing spline models assessed associations between anemia burden (YLDs) and the Socio-demographic Index (SDI) across 21 regions and 204 countries/territories (16).

SDI, quantifying national development (0 = least, 1 = most developed), integrates lag-distributed income per capita, decade-smoothed GDP per capita, educational attainment among ≥15-year-olds, and under-25 total fertility rate. Spatial mapping of age-standardized prevalence and YLD rates employed R software version 3.5.2.

### The auto-regressive integrated moving average model

The Autoregressive Integrated Moving Average (ARIMA) model is a statistical tool employed for forecasting time series data. It integrates three primary elements: (1) Autoregression (AR), which describes the correlation between a data point and a specified number of preceding data points, assuming that the value of a variable is a linear combination of its past values; (2) Differencing (I), which involves transforming a non-stationary time series into a stationary one by eliminating trends and seasonal patterns. This is achieved by subtracting the current value from the previous value; and (3) Moving Average (MA), which represents the time series as a constant plus the mean of a certain number of error terms from prior observations, capturing the influence of random fluctuations or residuals on the current value.

The ARIMA model is represented as ARIMA (p, d, q), where p denotes the number of autoregressive terms, d indicates the degree of differencing required to achieve stationarity, and q specifies the order of the moving average. This model is particularly effective for datasets with a consistent mean and variance over time, characteristic of stationary data. It is extensively utilized in fields such as economics and finance for predicting future values based on historical observations. However, it is important to recognize that ARIMA assumes a linear and Gaussian nature of the underlying data generation process, which may not always align with real-world complexities.

### Statistical analysis

Data were displayed as estimates accompanied by a 95% confidence interval (CI). Age-standardized rates of years lived with disability (YLDs) were quantified per 100,000 individuals. For non-normally distributed data, the Kruskal-Wallis test was utilized to assess disparities in age-standardized rates between genders. The autoregressive integrated moving average (ARIMA) model, a prevalent tool in time series forecasting, was employed to project the burden of vision loss due to diabetes from 2021 to 2045 (R system, version 4.2.2; detailed methodology provided in Supplementary materials). Unless otherwise stated, all statistical analyses were performed using Prism software Version 9.0 (GraphPad, San Diego, California) and the Echarts open-source platform. Statistical significance was set at a *p* value of less than 0.05.

## Result

### Burden of anemia caused by NCDs from 1990 to 2021

**Figure 1:**
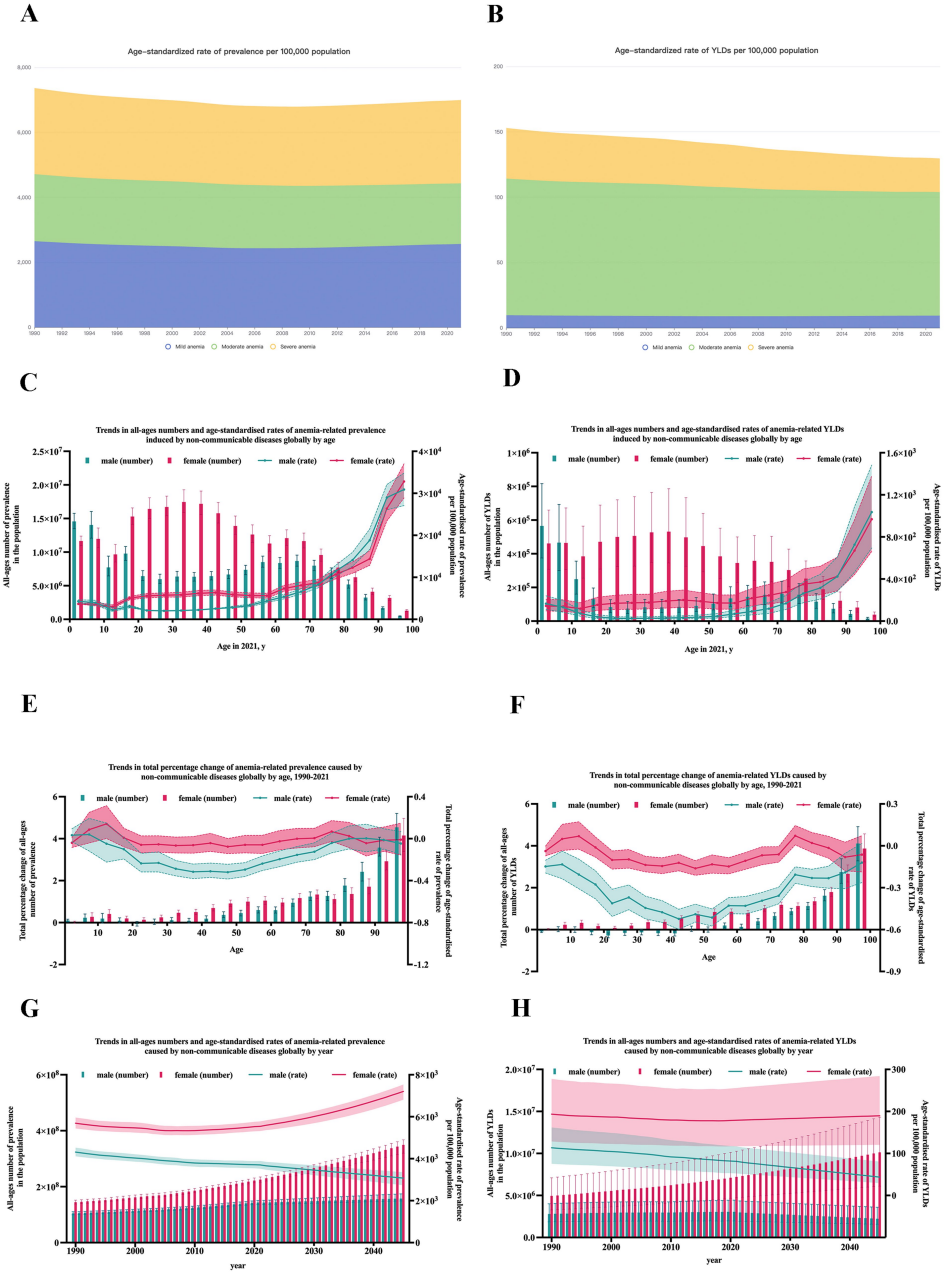
The trends of the burden of anemia caused by NCDs globally. **(A)** Age-standardized rate of prevalence by year and severity; **(B)** Age-standardized rate of YLDs by year and severity; **(C)** Prevalence by age; **(D)** YLDs by age; **(E)** Annual rate change of prevalence by age; **(F)** Annual rate change of YLDs by age; **(G)** Prevalence by year; **(H)** YLDs by year; Error bars indicate the 95% uncertainty interval (UI) for numbers. Shading indicates the 95% UI for rates.

The global burden of anemia caused by NCDs has seen a significant increase from 1990 to 2021. Globally, the all-ages prevalence number surged from 2491.40 million (95% uncertainty interval [UI]: 2375.72–2604.15) in 1990 to 3684.10 million (95% UI: 3531.96–3845.99) in 2021, marking a 47.9% increase over the past 30 years. Meanwhile, the age-standardized prevalence rate was 4982.4 (95% UI: 4759.0–5200.4) per 100,000 population in 1990 and 4608.5 (95% UI: 4414.8–4813.8) per 100,000 population in 2021. The number of YLDs in 1990 was 77.42 (95% UI: 51.32–110.94) million, increased to 102.28 (95% UI: 3.63–7.36) million, with an annual growth rate of 1.04%. The age-standardized rate of YLDs in 1990 was 152.9 (95% UI: 101.5–218.4) per 100,000 population and decreased to 129.6 (95% UI: 84.5–185.3) in 2021 (Figures 1G,H; Supplementary materials).

According to severity, anemia was divided into three levels: mild anemia, moderate anemia, and severe anemia. The prevalence of anemia caused by NCDs cases [mild: 2074.80 (95% UI: 1984.60–2169.10) million; moderate: 1468.60 (95% UI: 1404.36–1539.63) million; severe: 140.60 (95% UI: 132.74–148.69) million], and age-standardized rate (per 100,000 population) [mild: 2566.3 (95% UI: 2452.2–2682.1); moderate: 1863.7 (95% UI: 1779.8–1955.4); severe: 178.5 (95% UI: 168.3–189.0)]. In addition, the number of YLDs [mild: 7.59 (95% UI: 2.71–16.74) million; moderate: 74.40 (95% UI: 48.01–107.50) million; severe: 20.28 (95% UI: 14.00–28.38) million], and age-standardized rate (per 100,000 population) [mild: 9.4 (95% UI: 3.4–20.8); moderate: 94.5 (95% UI: 60.9–136.4); severe: 25.8 (95% UI: 17.8–36.1)] (Figures 1A,B; Supplementary materials).

### Burden of anemia caused by NCDs by age and sex

With age, both the global numbers of anemia-related prevalence and YLDs exhibits a double-peaked curve, with peaks occurring at children under five and women of reproductive age. While the age-standardized rates of anemia-related prevalence and YLDs gradually increased with age (Figures 1C,D). As the anemia burden also increased with time, to observe the growth rate in different age groups, percent change of burden was calculated to represent the annual rate of growth. The annual rate of growth of prevalence and YLDs increased with age (Figures 1E,F).

As shown in Figures 1C–F, the number of anemia prevalence and YLDs in females were constantly higher than that in males from 1990 to 2021. But for age groups, notably, an intersection in the curves of different genders around age 14, suggesting women bear a heavier burden of anemia-related issues compared to men after the age at which females experience menarche. For age-standardized rates of anemia cases and YLDs, the trends stayed invariably, from 1990 to 2021, in all age groups. It’s worth noting that with time and age, the difference between two genders grew obviously dramatic.

### Future prediction in the burden of anemia caused by NCDs

Based on the trend observed, the ARIMA model was applied to project the future trend to 2045. As estimated, as shown in Figures 1G,H, in 2045, there will be about 157.74 (95% UI: 148.80–174.22) million male cases and 349.56 (95% UI: 319.97–367.32) million female cases. And the prevalence rate per 100,000 population will reach approximately 3077.0 (95% UI: 2736.3–3360.6) in males and 7208.5 (95% UI: 6812.2–7532.7) in females, respectively. And the YLDs rate will reach approximately 43.7 (95% UI: 29.5–80.6) in males and 189.7 (95% UI: 120.6–284.3) in females.

### Burden of anemia caused by NCDs by countries and territories

**Figure 2:**
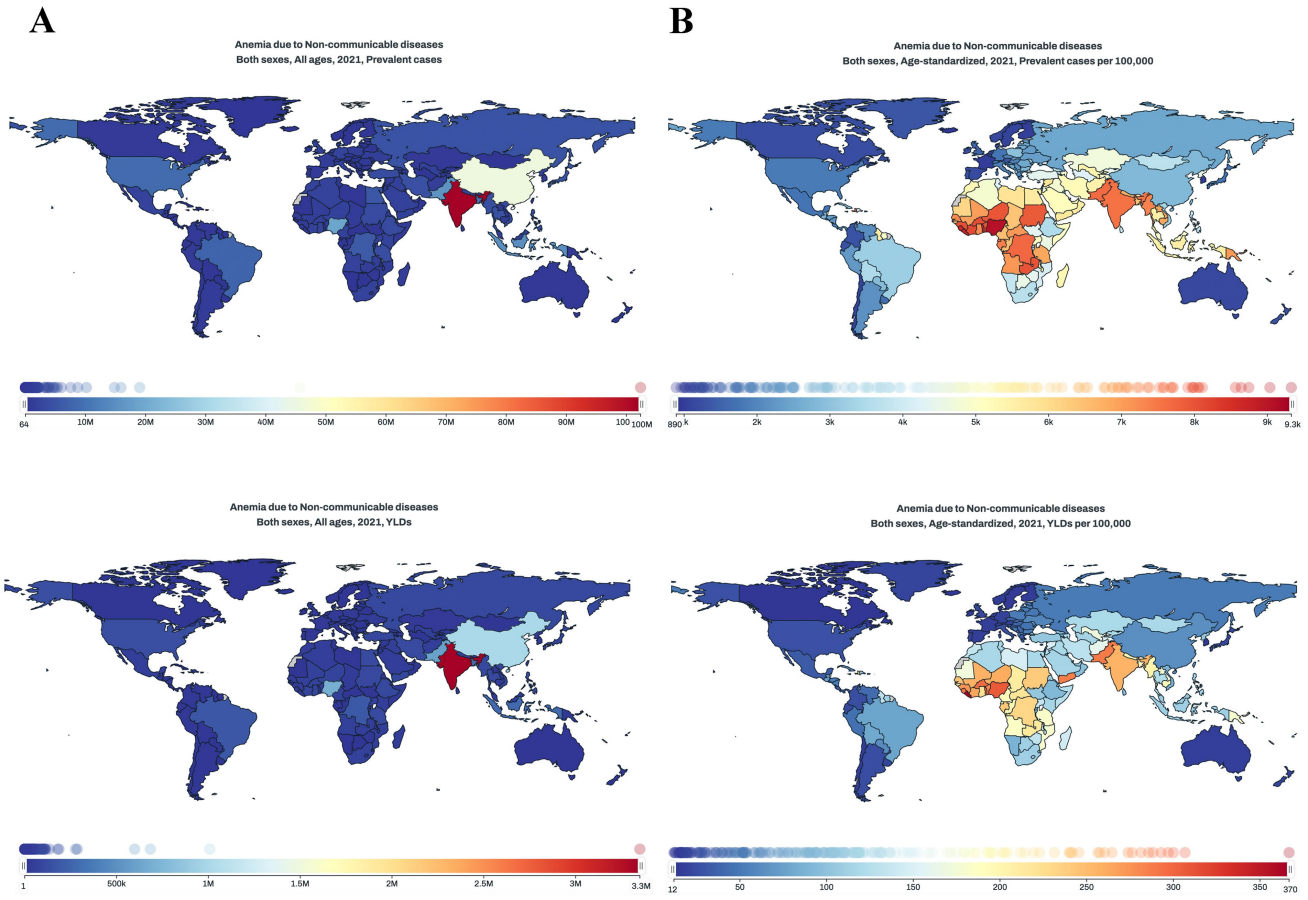
The heat-map of the burden of anemia caused by NCDs. **(A)** Prevalence; **(B)** YLDs.

**Figure 3:**
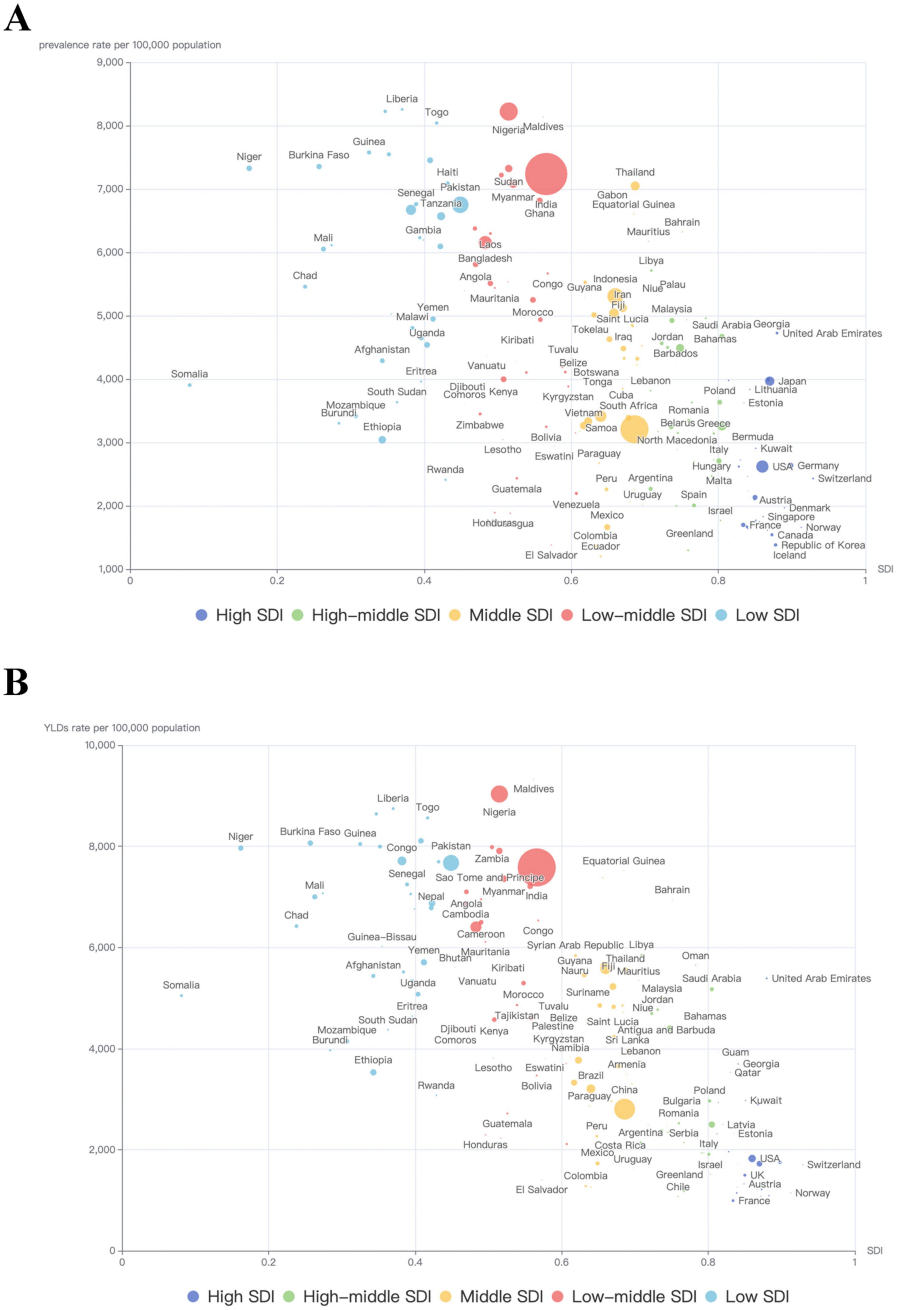
The burden of anemia caused by NCDs in 204 countries by SDI groups in 2021. **(A)** Prevalence; **(B)** YLDs. The area of bubbles represents all-ages number of HF-related burden.

In 2021, among the 204 countries and territories, the top 3 largest number of anemia cases caused by NCDs occurred in India: 102.35 million (95% UI: 97.49–108.13), China: 45.63 million (95% UI: 42.15–49.89), Nigeria: 19.01 million (95% UI: 17.08–20.96). The top 3 countries with highest anemia prevalence rate were Maldives: 9328.0 (95% UI: 7769.9–11013.2), Nigeria: 9033.8 (95% UI: 8249.3–9856.7), Liberia: 8744.7 (95% UI: 7455.1–10379.2). In contrast, Iceland [891.4 (95% UI: 782.5–1019.4)], Republic of Korea [956.3 (95% UI: 823.2–1089.9)] and France [991.3 (95% UI: 876.8–1131.7)] had the lowest age-standardized rate (per 100,000 population). Same for YLDs, the highest numbers were observed in India [3.35 million (95% UI: 2.21–4.75)], China [1.01 million (95% UI: 0.65–1.48)], and Nigeria [0.68 million (95% UI: 0.44–0.97)]. Liberia [366.7 (95% UI: 238.1–528.7)], Nigeria [306.7 (95% UI: 201.8–435.6)], Togo [300.1 (95% UI: 194.3–424.9)] had the highest age-standardized rate (per 100,000 population). Monaco [12.0 (95% UI: 7.2–19.1)], Republic of Korea [12.5 (95% UI: 7.8–18.9)] and Canada [12.6 (95% UI: 7.0–22.3)] had the lowest age-standardized rate (per 100,000 population) (Figures 2, 3).

### Burden of anemia by different NCDs

**Figure 4:**
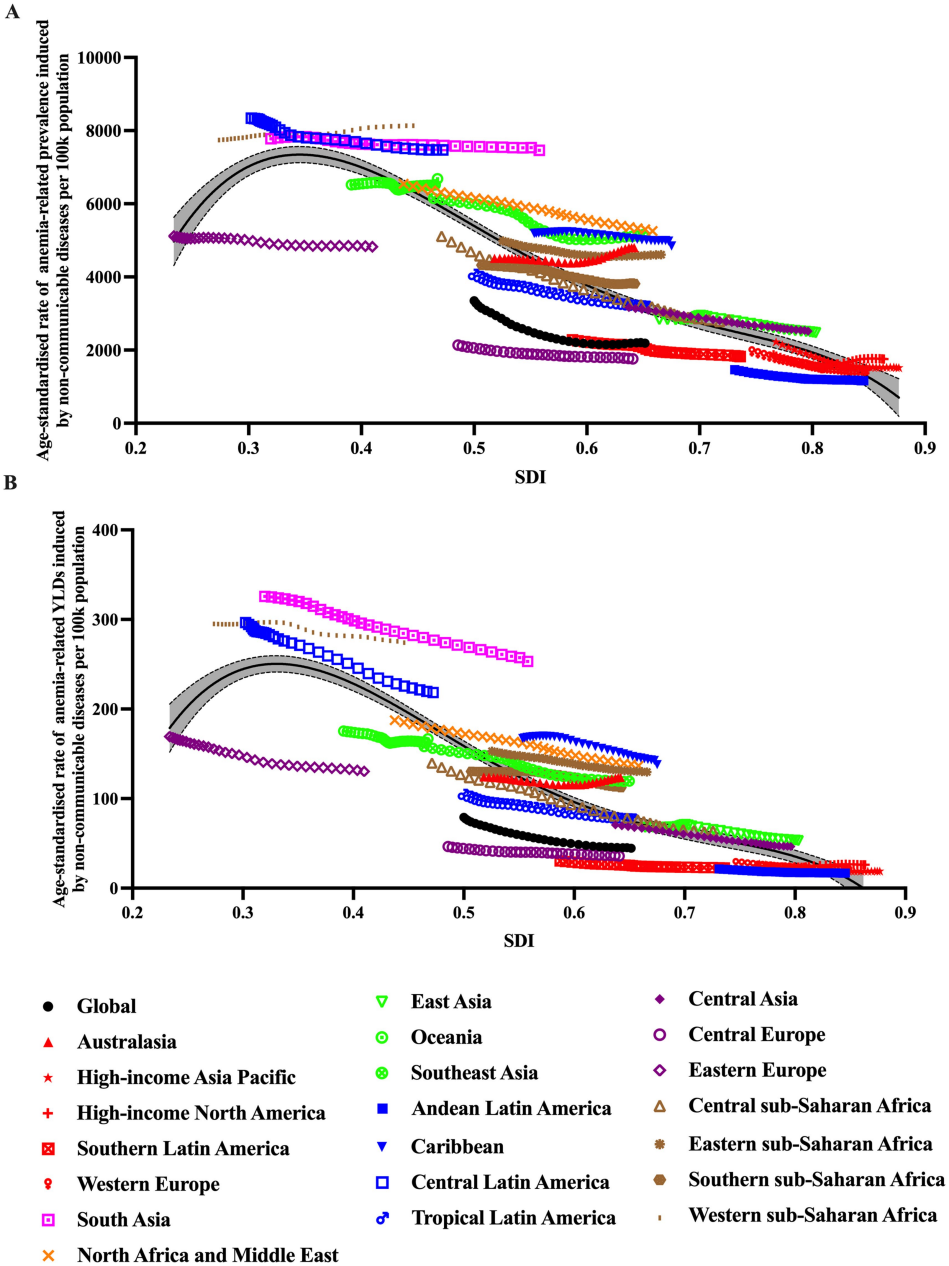
The burden of anemia caused by NCDs in different super regions by SDI. **(A)** Prevalence; **(B)** YLDs.

In 2021, the disease with the highest observed age-standardized prevalence and YLDs rate is hemoglobinopathies and hemolytic anemias, followed by chronic kidney disease (CKD), and endocrine, metabolic, blood, and immune disorders. Hemoglobinopathies and hemolytic anemias, being the key underlying cause of anemia, accounted for 69.0% of the anemia-related burden, with 252.8 (95% UI:239.7–267.4) million in all-ages prevalence, and 7.03 (95% UI:4.58–9.98) million in all-ages YLDs, showing an increase of 38.6 and 23.1% from 1990. Meanwhile, the age-standardized prevalence rate was 3221.7 (95% UI: 3052.3–3408.9) per 100,000 population, and the age-standardized YLDs rate was 91.1 (95% UI: 59.4–129.6) per 100,000 population, decreased by 6.3 and 14.1% since 1990 (Figure 4).

**Figure 5:**
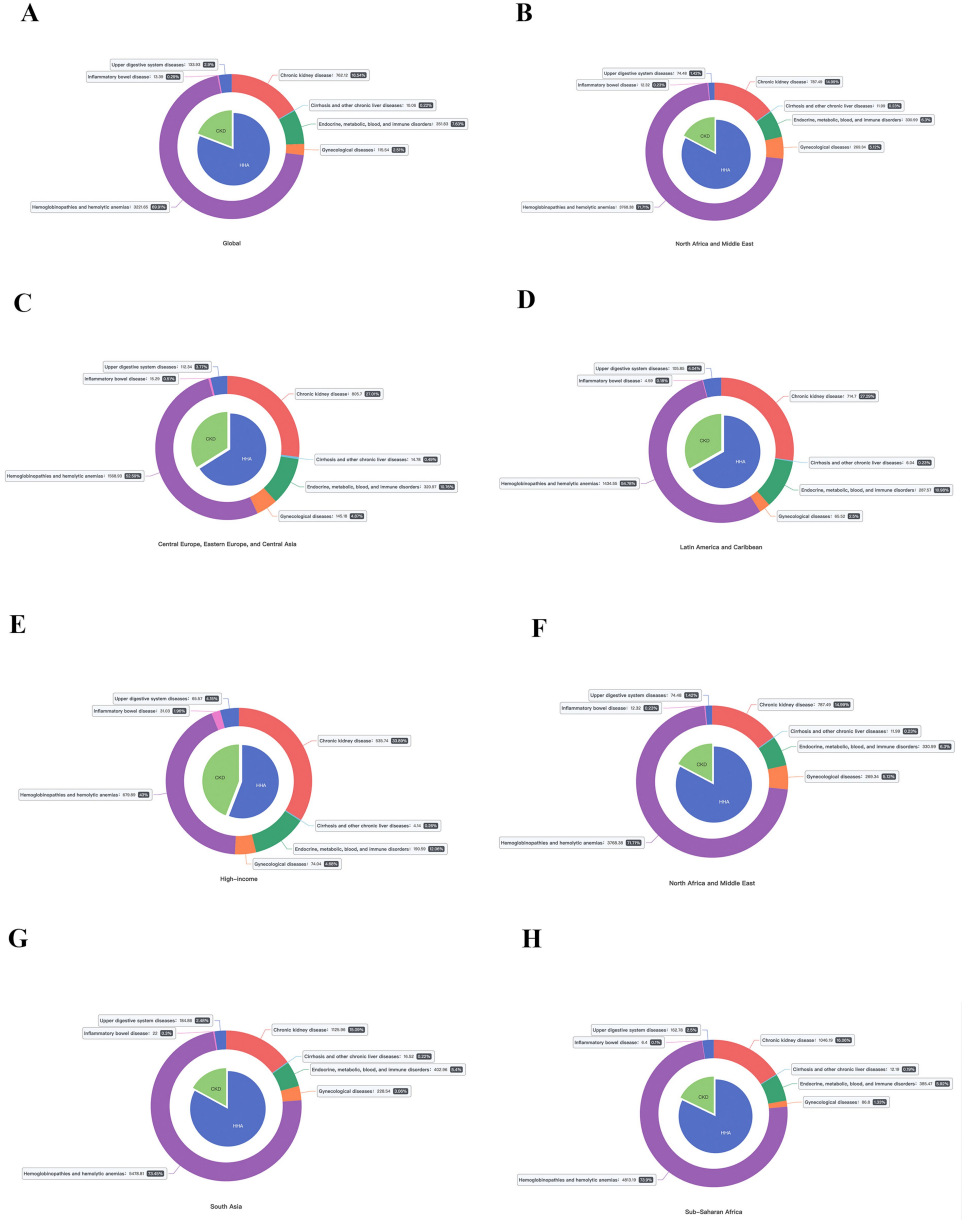
The proportion of the burden of anemia caused by different NCDs in super regions.

In terms of the total percentage change of anemia-related burden, the top three underlying causes that increased the most were CKD, inflammatory bowel disease (IBD), and gynecological diseases, rising by 96, 71, and 67%, respectively. In nearly all GBD regions, hemoglobinopathies and hemolytic anemias was the leading cause, while in High-income Asia Pacific, High-income North America and Western Europe, it was CKD (Figure 5).

### Burden of anemia caused by NCDs by SDI and health system grouping levels

**Figure 6:**
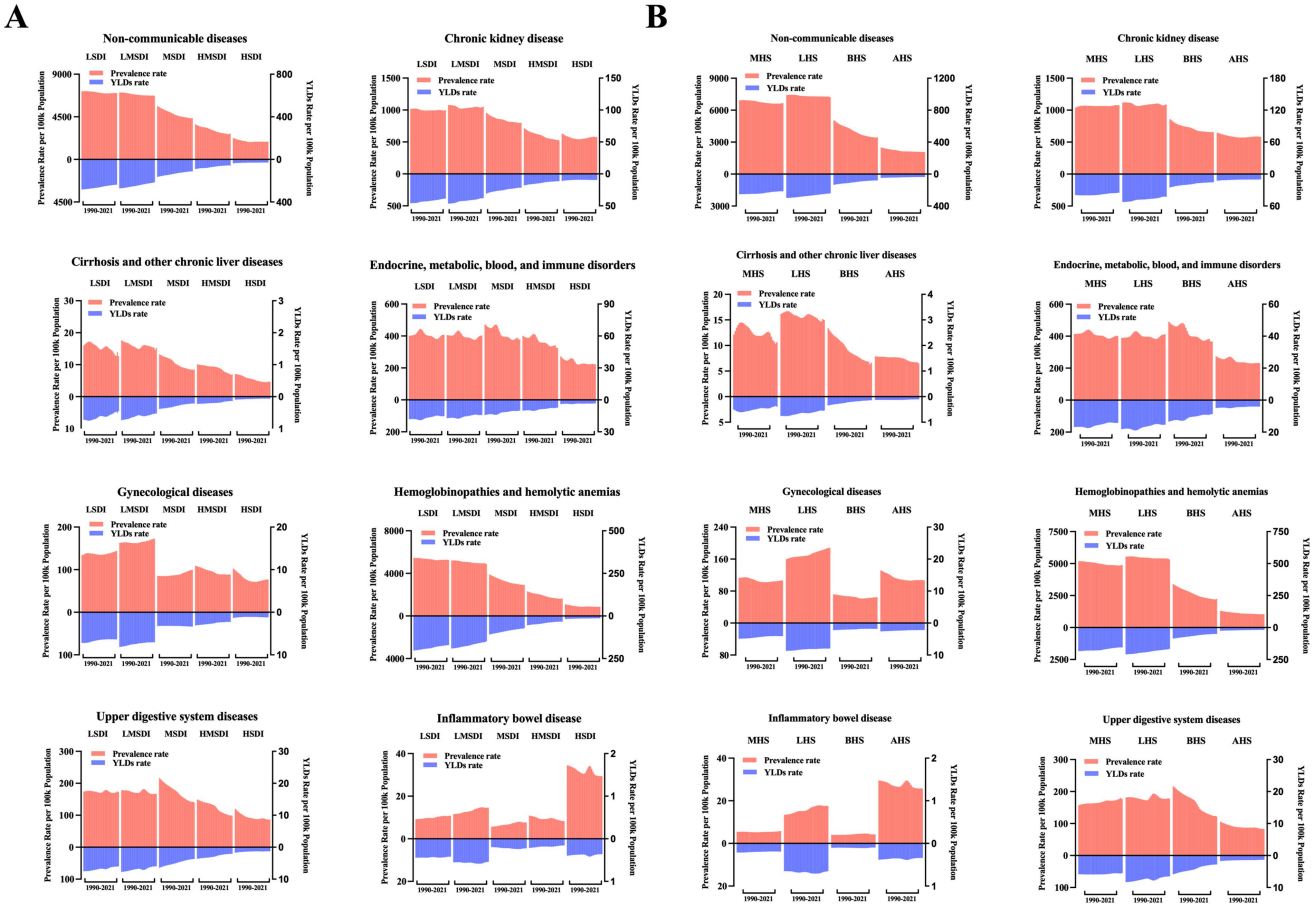
The burden of anemia caused by different NCDs by year. **(A)** SDI groups; **(B)** Health system grouping levels.

Figure 6A showed the age-standardized prevalence rate and YLDs rate per 100,000 population of anemia by SDI and leading causes. It seems there was an association between SDI and prevalence rate and YLD rate, respectively. First, in all SDI subgroups, both the prevalence rate and YLDs rate had an evident trend of decreasing over time, from 1990 to 2021. And the highest prevalence rates and YLDs rates always appeared in low-middle and middle SDI regions.

In GBD study 2021, it additionally provided information of health system grouping levels by location. Generally, the trend by health system grouping level was similar with that of SDI: from 1990 to 2021, the burden of anemia decreased in all levels of health system groups (Figure 6B); the heaviest burden appeared in countries with limited health system, and the lowest rates presented in countries with advanced health system.

## Discussion

The interplay between anemia and non-communicable diseases (NCDs) presents a complex and multifaceted challenge to global health, with significant implications for disease burden and management. According to the Global Burden of Disease Study 2021 (GBD 2021), anemia remains a prevalent condition worldwide, particularly among populations affected by NCDs such as diabetes, cardiovascular diseases, and chronic kidney disease. The coexistence of anemia with these NCDs exacerbates morbidity and mortality rates, as anemia impairs oxygen delivery to tissues, leading to reduced organ function and overall physical capacity.

Economic analyses reveal that anemia imposes substantial costs on healthcare systems and economies. The direct costs include expenses related to diagnostic tests, treatments, and hospitalizations, while indirect costs are associated with reduced productivity and lost workdays. In patients with NCDs, the presence of anemia often leads to more severe disease progression and higher healthcare utilization, further increasing the economic burden. Gender and regional disparities in anemia prevalence are also evident. Females, particularly those in rural areas, are disproportionately affected due to factors such as nutritional deficiencies and limited access to healthcare. Geographical variations highlight higher anemia rates in regions with lower socioeconomic status, where malnutrition and inadequate healthcare infrastructure are prevalent. Interventions aimed at managing anemia in the context of NCDs must be multifaceted, addressing both the underlying causes and the symptoms. Nutritional supplementation, particularly iron and vitamin B12, is crucial for addressing deficiency-related anemia. Additionally, managing the underlying NCDs through appropriate medical care and lifestyle modifications can help mitigate the impact of anemia. The influence of economic development and cultural practices on anemia burden is significant. Improved economic conditions can enhance access to nutritious food and healthcare, thereby reducing anemia rates. However, cultural practices, such as dietary habits and traditional beliefs, can either mitigate or exacerbate the risk of anemia, depending on their alignment with nutritional requirements.

In low-middle and middle SDI countries, anemia is a significant public health challenge. The prevalence of anemia in these regions is notably higher compared to high SDI areas. For instance, in 2021, the prevalence of anemia was highest in western sub-Saharan Africa (47.4%), followed by South Asia (43%) and central sub-Saharan Africa (35.7%) (11). The high prevalence in these regions can be attributed to multiple factors, including limited access to diverse and nutritious foods, inadequate healthcare systems, and the prevalence of infectious diseases such as malaria, which contribute to the anemia burden. Additionally, socio-economic factors play a crucial role, as higher SDI regions generally benefit from better living standards, healthcare access, and nutrition, which help mitigate the burden of anemia.

In children under 5 years, anemia remains a significant health concern. Despite global efforts, the burden of anemia in this age group remains high, with a global age-standardized YLDs attributable to anemia in children under five calculated at 1,252.88 per 100,000 in 2019 (17). The high rates of anemia in this age group can be attributed to rapid growth and development, which increase the demand for iron and other nutrients (18). Moreover, dietary habits in low-middle and middle SDI countries often lack sufficient iron-rich foods, exacerbating the problem. The impact of anemia in early childhood can lead to long-term cognitive and physical development impairments, affecting future health and productivity (5).

In women, anemia is more prevalent, especially during reproductive years. This is primarily due to menstrual blood loss and the increased iron demands of pregnancy. In 2021, 31.2% of women globally had anemia compared to 17.5% of men, with the gender difference being more pronounced during the reproductive years (ages 15–49) (19). The World Health Organization recommends a daily 30–60 mg elemental iron supplement for adult women and adolescent girls due to their high risk of deficiency (20). However, despite these recommendations, the prevalence of anemia in women of reproductive age remains a significant challenge, indicating the need for more integrated efforts in areas where anemia is more prevalent (9).

In regions with low-middle SDI, where anemia is a prevalent health concern, a variety of strategies have proven effective in enhancing treatment rates. These approaches tackle the complex interplay of nutritional, healthcare, and socio-economic dimensions of anemia:

1. Nutritional Strategies: Iron deficiency stands out as a primary driver of anemia, particularly affecting children and women. Consistent iron supplementation programs have demonstrated efficacy. The World Health Organization advocates for daily iron supplementation for women and adolescent girls to combat iron deficiency anemia. In low-middle SDI settings, leveraging community health workers and local clinics for iron supplement distribution markedly boosts accessibility and compliance (21). Additionally, fortifying staple foods with iron and other micronutrients is a potent strategy, ensuring a broad population receives vital nutrients via their regular diet. For example, iron-fortified flour has been instrumental in reducing anemia in multiple countries (22).
2. Healthcare Infrastructure Enhancement: Boosting the diagnostic capabilities of healthcare facilities is pivotal. This entails equipping facilities with necessary tools and training staff to conduct and interpret blood tests accurately. Mobile health units can extend diagnostic services to remote areas in low-middle SDI countries. Furthermore, integrating anemia management into existing healthcare frameworks, like maternal and child health services, can elevate treatment rates. For instance, embedding anemia screening and treatment within prenatal and well-child visits ensures continuous monitoring and treatment for high-risk populations (1).
3. Public Health Initiatives: Increasing awareness of anemia’s causes and impacts is fundamental. Public health campaigns can inform communities about the value of a balanced diet, the advantages of iron supplementation, and the necessity of regular health assessments. Such campaigns can be executed through local media, community gatherings, and educational institutions. Promoting behaviors that mitigate anemia risk, like consuming iron-rich foods and maintaining hygiene to prevent infections, is also effective. For example, encouraging the intake of green leafy vegetables and fortified products can address nutritional shortfalls (22).
4. Focused Interventions for Vulnerable Groups: Pregnant women and children under 5 are especially susceptible to anemia. Tailored programs targeting these groups can make a significant difference. For example, providing iron and folic acid supplements to pregnant women and ensuring children receive routine health check-ups and nutritional support can lower anemia rates. Health education and nutritional support for adolescent girls can preempt chronic anemia. School-based initiatives offering iron supplements and education on menstrual health and nutrition have proven effective (23).
5. Policy and Regulatory Frameworks: Governments can significantly impact anemia reduction by crafting and executing national health policies. These may encompass mandatory food fortification, subsidized iron supplements, and enhanced healthcare infrastructure. Collaborations with NGOs and international bodies can bring additional resources and expertise. For instance, the Bill & Melinda Gates Foundation has backed several initiatives aimed at reducing anemia in low-middle SDI regions (24).

This secondary examination of data from the GBD 2021 study provides a comprehensive assessment of anemia attributable to NCDs on a global and national scale, across various dimensions such as time, age, gender, and SDI. Utilizing the most recent data updates and employing standard methodologies, this analysis also projects future trends. However, several limitations must be acknowledged. Firstly, the quality of data within the GBD is variable, particularly in less developed regions where population-based data are scarce. Secondly, disparities in health information systems contribute to gaps in data consistency. Thirdly, many factors are interrelated, complicating the isolation of individual causal effects. Lastly, there is a notable absence of data concerning socio-cultural or racial contexts and the diverse phenotypes of anemia.

In conclusion, the burden of anemia caused by NCDs in low-middle and middle SDI countries, in children under 5 years, and in women is a complex issue influenced by a combination of nutritional, socio-economic, and health system factors. Addressing this burden requires a multifaceted approach, including improving access to nutritious foods, enhancing healthcare systems, and implementing targeted public health interventions to reduce the prevalence and impact of anemia.

## Data Availability

The original contributions presented in the study are included in the article/Supplementary material, further inquiries can be directed to the corresponding authors.
